# Effect of fluoride, chlorhexidine or Nd:YAG on the progression of root dentin demineralization after removal of the demineralized organic matrix *

**DOI:** 10.1590/1678-7757-2021-0496

**Published:** 2022-03-11

**Authors:** Andrea Maselli, Tânia Mara da Silva, Lucélia Lemes Gonçalves, Aline Silva Braga, Eduardo Bresciani, Ana Carolina Magalhães, Sérgio Eduardo de Paiva Gonçalves

**Affiliations:** 1 Universidade Estadual Paulista Instituto de Ciência e Tecnologia de São José dos Campos Departamento de Odontologia Restauradora São José dos Campos SP Brasil Universidade Estadual Paulista (UNESP), Instituto de Ciência e Tecnologia de São José dos Campos, Departamento de Odontologia Restauradora, São José dos Campos, SP, Brasil.; 2 Faculdade Anhanguera Educacional Faculdade de Odontologia São José dos Campos SP Brasil Faculdade Anhanguera Educacional, Faculdade de Odontologia, Campus São José dos Campos, São José dos Campos, SP, Brasil.; 3 Universidade de São Paulo Faculdade de Odontologia Departamento de Ciências Biológicas Bauru SP Brasil Universidade de São Paulo (USP), Faculdade de Odontologia, Departamento de Ciências Biológicas, Bauru, SP, Brasil.

**Keywords:** Dentin, Demineralization, Collagen-degradation, Hydroxyproline, Root caries

## Abstract

Quantification of collagen degradation is an important parameter to evaluate dentin caries for preventive aid.

**Objectives:**

Evaluate preventive methods against root collagen degradation by the hydroxyproline assay (HYP) and microradiography technique (MRT).

**Methodology:**

Five bovine root dentin blocks were obtained and subjected to an artificial demineralization process by acetate buffer (pH 5) to induce carious lesion formation. Samples were subjected to the following therapeutic treatments: 1) 0.12% chlorhexidine for 1 min, 2) 2% fluoride for 1 min, 3) Nd:YAG Laser (400 μm diameter optical fiber, 10 Hz frequency, 60 mJ/pulse energy, 48 J/cm2 energy density, in noncontact mode for 10 s), 4) deionized water (control) for 1 min, 5) MRT control group (without treatment and removal of collagen). Samples were exposed to degradation by a collagenase enzyme for five days. The enzyme solution was collected, by colorimetry in a spectrophotometer, from the collagen matrix for the hydroxyproline release analysis. The same samples were subjected to an additional two days of demineralization to induce the progression of mineral loss. Samples were analyzed by MRT for the visualization of their degraded areas (estimation of lesion depth and mineral loss). ANOVA was applied to compare hydroxyproline release rates. MRT data were subjected to the Kruskal-Wallis test, followed by the Dunn’s test. Comparisons between the initial five-day and the subsequent two-day demineralization processes were performed by repeated t-test or Wilcoxon (p<0.05) measurements.

**Results:**

The amount of HYP released from the dentin samples failed to show significant differences among the groups (p=0.09). Fluoride and chlorhexidine were able to interact with the samples, reducing the progression of dentin caries after removal of the demineralized organic matrix. CHX was the only treatment able to show significant lower lesion depth than the negative control.

**Conclusion:**

Chlorhexidine and fluoride were effective in reducing root caries progression.

## Introduction

As advances in dentistry have led to significant improvements to the population’s oral health, people not only live but also retain their own teeth longer.^1^ This has been inevitably associated with the frequent detection of root exposure due to gingival recession and, consequently, the development of root carious lesions affecting older adults.^2^ Preventing these situations saves time and money and benefits individuals’ quality of life.

Root carious lesions develop as a consequence of mineral loss associated with collagenolytic degradation.^3^ Once demineralized, the superficial dentin organic matrix is exposed, becoming susceptible to enzymatic degradation.^4^ The degradation of the demineralized organic matrix (DOM) can increase the progression of dentin carious lesions.^5^ This process occurs by the activity of host enzymes, such as matrix metalloproteinases (MMPs) and cysteine cathepsins (CCs), present in saliva and dentin. Such enzymes are activated in acidic pH and degrade the exposed collagen, as with CCs and MMPs in acidic and neutralized pH, respectively.^3^

Theoretically, if the collagen fibril scaffold of the DOM is preserved and appropriate mineral supplementation is provided, dentin remineralization may occur. Therefore, inhibiting its degradation may be of interest to avoid the progression of dentin caries.^6^ Different agents (i.e., fluoride, chlorhexidine, and laser) have served as prevention by reducing mineral loss and/or DOM degradation by physical and chemical changes on its substrate.^5 , 7^

Fluoride (F) plays an important role in the control of root carious lesions by reducing caries progression rates and inducing the arrest of active lesions.^8^ Professional fluoride products, applied on cleaned surfaces, allow the precipitation of globule-like CaF_2_ on the tooth surface, which acts as a mechanical barrier and F reservoir to interact with teeth during demineralization-remineralization processes.^9^

Chlorhexidine (CHX) is known to have antimicrobial effect and to protect the DOM against degradation^5^ with the potential to control root dentin caries.^10^ The literature has reported that CHX reduces the self-degradation of collagen fibrils by inhibiting host-derived protease activity (MMPs and CCs) in demineralized dentin.^7,11-13^

High power laser irradiation can also inhibit demineralization, if applied under specific parameters such as wavelength, density, pulse width, and repetition rate. Studies have found that Nd:YAG laser (1064 nm) enhances the acid-resistance of dental hard tissues by modifying their chemical and physical structure.^14^ This modification includes melting, carbonate reduction, and α- or β-tricalcium phosphate and tetracalcium phosphate formation, which are less soluble than hydroxyapatite.^14^ It also changes the bands attributed to the collagen matrix.^15^ However, even though Nd:YAG has been tested for caries control, controversial results have been reported on its effects on dentin.^16^ Furthermore, concerns about heating, photoabsorber presence, and correct laser parameters are still under investigation.^14 , 17 , 18^

Considering the three possible mechanisms of action (reducing mineral loss, preventing DOM degradation, and/or promoting physical and chemical changes on the substrate), this study aimed to investigate the effect of 2% NaF (professional application), 0.12% CHX, and Nd:YAG laser irradiation (60 mJ) on the progression of dentin demineralization when the DOM is subjected to enzymatic degradation. We performed a hydroxyproline assay (HYP) to analyze DOM degradation (HYP), and transverse microradiography (MRT), to measure lesion progression. Our null hypotheses were: 1) our treatments fail to reduce or avoid DOM degradation (measured by HYP assay) when compared to a negative control, and 2) our treatments fail to reduce dentin mineral loss progression (measured by MRT) when compared to a negative control.

## Methodology

### Sample preparation

This study was approved by the local Institutional Review Board (protocol number 06/2017). In total, 50 bovine incisors were selected, cleaned, and immersed in deionized water until use. Tooth crowns were sectioned 2 mm from the cementoenamel junction with a diamond wheel (Dremel, Campinas, SP, Brazil). The roots were placed in a universal cutting machine with a trephine diamond bur to obtain round dentin samples measuring 6 mm in diameter and 1.5 mm in thickness. Samples were polished in a polishing machine (DP-10 Panambra, SP, Brazil) using 120, 600, and 1200 grit silicon carbide (SiC) papers (Extec. Corp. Erios, São Paulo, SP, Brazil) under refrigeration. All samples were individually stored in microtubes containing deionized water at 4ºC.

**Figure 1:**
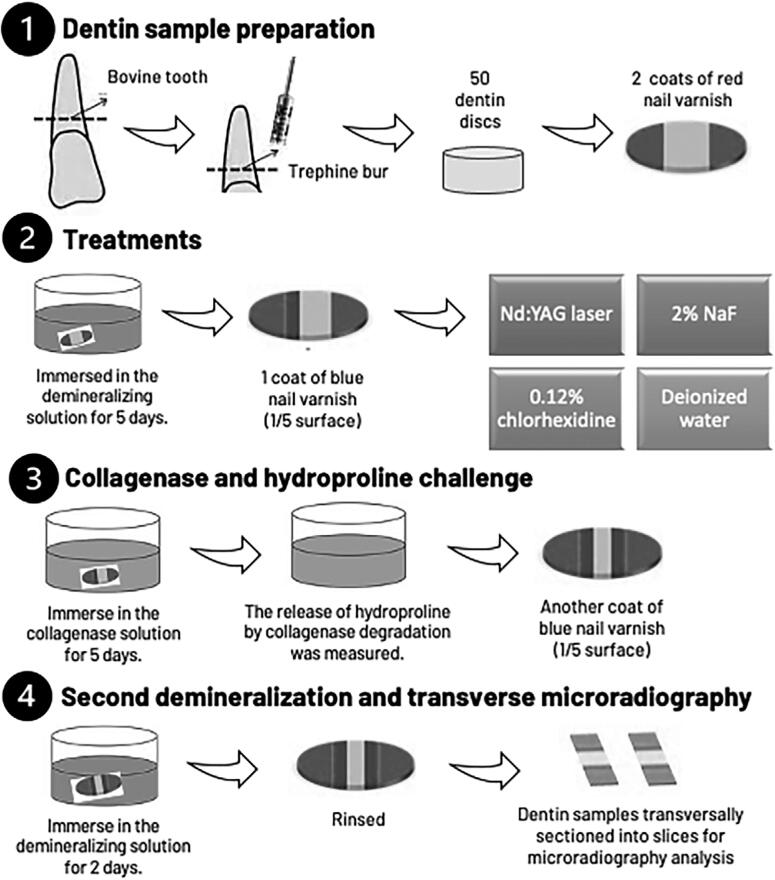
Experimental design of the study

The polished tooth surfaces were subjected to artificial caries formation. First, two coats of red nail varnish (Colorama Maybelline, São Paulo, SP, Brazil) were applied on the samples. Sample surfaces were divided into five parts, two of which were covered with nail varnish to act as a control area ( Figure 1 [1]).

Dentin samples were then subjected to a demineralization solution to create an incipient lesion, according to the protocol reported by Islam, et al.^19^ (2012). Samples were demineralized by an acetate buffer (0.1 mol/L; pH 5.0, v = 30 ml/sample) for five days at 37°C.

### Treatments

After demineralization, one coat of blue nail varnish was applied to another 1/5 of the sample surface to protect the demineralized area ( Figure 1 [2]). Samples were randomly divided into groups, according to treatment (n=10):^7 , 20^

- 2% NaF gel (9000 ppm F, pH 7.0, Fórmula e Ação, São José dos Campos, SP, Brazil). Fluoride was applied directly on the top surface of samples with an applicator tip (Microbrush KG Sorensen) for 1 min. Next, samples were cleaned with absorbent paper.

- 0.12% chlorhexidine solution (Fórmula e Ação, São José dos Campos, SP, Brazil). The solution was applied directly on the top surface of samples with an applicator tip (Microbrush KG Sorensen) for 1 min. Next, samples were cleaned with absorbent paper.

- Nd:YAG laser (Pulse Master 600 IQ, American Dental Technologies, TX USA). Surfaces were irradiated with an Nd:YAG laser set at 1064 nm wavelength, 400 μm diameter optical fiber, 10 Hz frequency, with 60 mJ/pulse energy and 48 J/cm^2*^ energy density. Laser irradiation was performed in noncontact mode, and surfaces were scanned for 10 seconds by the same calibrated operator. During laser irradiation, the fiber tip was positioned perpendicularly to sample surfaces at a distance of approximately 1 mm, supported by an acrylic resin device.

- Negative control group: Deionized water was applied on surfaces for 1 min. Next, samples were cleaned with absorbent paper.

- MRT control group: no treatment and no collagen removal.

### Collagenase and hydroxyproline challenges

After the treatment protocols, dentin samples (except the MRT control group) were subjected to collagen degradation by the action of collagenase ( Figure 1 [3]). The collagenase enzyme, obtained from *Clostridium histolyticum* (Type VII, Product No. C0773, Sigma-Aldrich, St. Louis, MO, USA), and added in artificial saliva (20 mmol/l HEPES, 0.70 mmol/l CaCl_2_, 0.20 mmol/l MgCl_2_.6 H_2_O, 4 mmol/l KH_2_PO_4_, 30 mol/l KCl, 0.30 mmol/l NaN_3_), contained 100 U/ml of an EDTA-free protease inhibitor cocktail (Complete^TM^ Protease Inhibitor Cocktail, Roche).^26^ Each sample was placed in the collagenase solutions (1.5 ml/sample) for 5 days at 37° C.

Hydroxyproline (HYP) release by collagen degradation was measured via the chloramine-T method (Hydroxyproline Assay Kit, Product No. MAK008, Sigma-Aldrich, St. Louis, MO, USA). A standard curve was created by using a hydroxyproline standard solution (0; 0.2; 0.4; 0.6; 0.8; and 1.0 μg HYP).

The enzyme solutions containing the degraded collagen were hydrolyzed by mixing 100 μl of the collagenase solution with 100 μl of ~ 12 M HCl, at 120ºC for 3 h. Activated charcoal (5 mg) were added to each glass tube, mixed and centrifuged at 13.000 x g for 2 min (Concentrator Plus, Eppendorf AG, Hamburg, Germany). Supernatants (50 μl) were transferred to a 96-well plate. For the reaction, 6 μl of chloramine-T into 96 μl of buffered solution were added to each well (samples and standards) and incubated at room temperature for 5 min. Next, 100 μl of the diluted DMBA (4-(Dimethylamino benzaldehyde)), which resulted in a colorimetric product, were added to each well and incubated for 90 min at 60°C. Absorbance was performed at 560 nm (Ultrospec 2000, Pharmacia Biotech, Cambridge, England), corrected from blank values, and converted into µg/mL.

After the collagenase challenge, samples were rinsed in deionized water and dried. One coat of blue nail varnish was applied to another 1/5 of the sample surface to protect the treatment area.

### Second demineralization and transverse microradiography (MRT)

After the HYP assay, specimens were further demineralized, as described above, for two more days to induce the progression of mineral loss. The experimental design of the study is shown in Figure 1 [4]. An MRT control group, only demineralized for five plus two days, without treatment and DOM removal, was included for comparisons.

All dentin samples were washed for 5 min in deionized water, sectioned transversally by low-speed diamond discs, and polished to obtain slices with 100-120 µm. These dentin slices were fixed into a sample-holder together with an aluminum calibration step wedge with 14 steps.

A microradiograph was taken by an X-ray generator (Softex, Tokyo, Japan) on a glass plate at 20 kV and 20 mA (at a distance of 42 cm) for 13 min. The glass slides were developed for 7 min, rinsed in deionized water, fixed for 7 min in a dark environment, and then rinsed in running water for 10 min and air-dried (all procedures were done at 20°C).

The developed plates were analyzed by a transmitted light microscope fitted with a 20x objective lens (Zeiss, Oberkochen, Germany), a CCD camera (Canon, Tokyo, Japan), and a computer. Two images per sample were taken using data-acquisition (version 2012), and the total area of the degraded collagen was measured by an image processing software (version 2006, Inspektor Research System, Amsterdam, Netherlands).

Mineral content was calculated according to the protocol reported by Angmar, et al.^21^ (1963), assuming mineral density to be 3.15 kgl^-1^ and 50 vol% of mineral content for the sound dentin. Lesion depth (LD, µm) and integrated mineral loss (∆Z, %vol.µm) were obtained.

### Statistical analysis

For all variables, assumptions of normal distribution and equality of variances were evaluated by the Kolmogorov-Smirnov and Bartlett’s tests, respectively. Since our assumptions were satisfied, the ANOVA and Tukey’s tests were applied to compare hydroxyproline release. Microradiography data were subjected to the Kruskal Wallis and Dunn’s tests for multiple comparisons. Demineralization progression was compared within each treatment group by the *t* -test or Wilcoxon tests. Significance level was set at 5%. The software GraphPad Prism (version 4.0 for Windows, San Diego, CA, USA) was used for the analyses.

## Results

### Hydroxyproline (HYP) data

**Figure 2:**
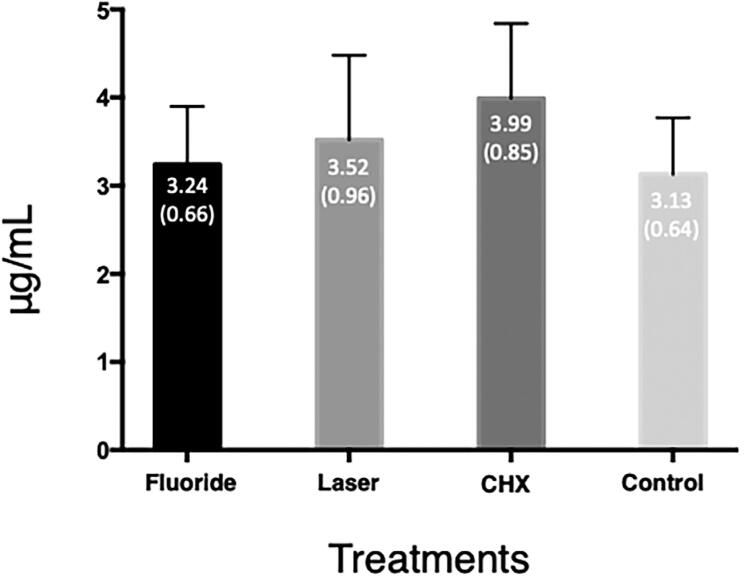
Amount of HYP (µg/mL) released from the dentin samples in each group

The amount of HYP released from the dentin samples failed to be significantly different among the groups, as Figure 2 shows (ANOVA, p=0.09).

### MRT data

We found that five-day demineralization produced a lesion with a mean 2485 (±699.6) %vol.μm mineral loss and a 114 (±42) μm lesion depth. Removal of the demineralized organic matrix failed to increase demineralization regardless of the treatment, which we expected since the protocol can only remove the unprotected collagen matrix rather than its mineral content.

**Figure 3:**
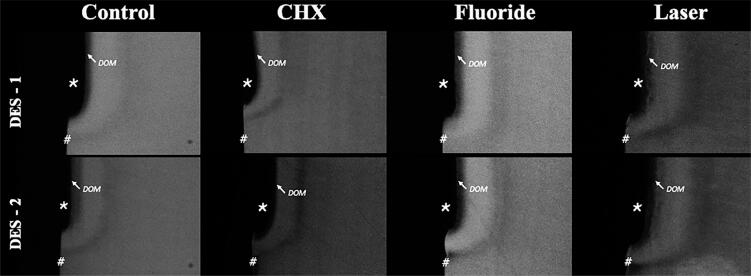
Representative MRT pictures of an enamel sample per group, including DES 1 (1st demineralization) and DES 2 (2nd demineralization after treatment) areas # sound area, * lost area, DOM - demineralized organic matrix. There is a mineralized area (radio space layer) below the DOM, followed by the front of demineralization (radiolucent layer)

Even though we found no differences among the treatments with respect to mineral loss after the 2^nd^ demineralization, further two-day demineralizing challenges induced the progression of mineral loss in dentin samples in the negative control and laser groups, whereas, mineral loss failed to progress in the other groups. This result shows that fluoride and chlorhexidine were able to interact with the teeth, reducing the progression of dentin demineralization after DOM removal ( Table 1 and Figure 3 ).

**Table 1:** Integrated mineral loss (ΔZ, %vol.μm) of dentin after different experimental conditions

	Fluoride	Laser	CHX	Negative Control	MRT Control	
	(without DOM)	(without DOM)	(without DOM)	(without DOM)	(with DOM)	
5d De+treatment (collagenase)	2967.5±496.6	2746.5±463.1	2819.5±435.6	2804.4±287.4	2485.0±699.6	ANOVA p=0.2957
5d De+treatment (collagenase)+2d De	3072.0±446.7	3196.5±457.5	2952.0±737.6	3546.0±259.5	3114.0±602.9	ANOVA p=0.1457
t-test (within the same treatment)	p=0.5954	p=0.0423	p=0.6307	p<0.0001	p=0.0229	
Mineral loss increase (yes/no)	no	yes	no	yes	yes	

With respect to lesion depth, we found significant differences among the groups after two additional days of demineralizing challenges. CHX was the only treatment able to show significant lower lesion depth than the controls (negative and MRT controls). Fluoride and laser treatments failed to differ from the controls. When we compared data within the same group, CHX was the only treatment which showed a decrease in lesion depth ( Table 2 ).

**Table 2:** Lesion depth (LD, μm) of dentin after different experimental conditions

	Fluoride	Laser	CHX	Negative Control	MRT Control	
	(without DOM)	(without DOM)	(without DOM)	(without DOM)	(with DOM)	
5d De+treatment (collagenase)	125.2±47.1	121.6±41.9	106.8±32.9	150.1±53.9	113.9±41.6	ANOVA p=0.2005
5d De+ treatment (collagenase)+2d De	102.0±20.6^ab^	98.2±18.0^ab^	78.3±17.2ᵃ	171.0±70.4^b^	128.6±43.7^b^	Kruskal Wallis/Dunn (p<0.0001)
t-test (within the same treatment)	p=0.4316*	p=0.2324*	p=0.0250	p=0.3527 p=0.0300$	p=0.1535	
Mineral loss increase (yes/no)	no	no	decreased	Yes (compared with DOM)	no	

## Discussion

In view of the increased prevalence of root caries lesions in older adults and the speed of caries progression in patients undergoing head and neck radiotherapy,^22 , 23^ this study discusses the effects of preventive procedures against root caries which employ possible chemical and/or physical changes promoted on the dental substrate. This is justified since the literature lacks full knowledge of the effects of fluoride, laser, and CHX on dentin demineralization and collagen degradation.^11 , 24 , 25^

Our model enabled us to assess the effects of the above preventive measures on dentin caries lesion progression. For this, we twice applied an abiotic model to simulate cariogenic challenges.^19^ Between both demineralizing challenges, we subjected specimens to treatments and DOM removal. To ensure data collection, the concentration of bacterial collagenase used for DOM removal in this study was much higher than that found in the oral cavity under clinical conditions, as per the literature.^26^

Fluoride, as already known, inhibits demineralization, increases remineralization,^27 , 28^ and limits the activity of microorganisms. to simulate a professional application,^27 , 29^ our fluoride concentration was 2% per minute. Though the literature lacks a full definition of its ideal value to prevent root cavities, systematic reviews on caries prevention have shown no differences among highly concentrated fluoride products.^30^

The second treatment elected for this study was chlorhexidine (CHX), considered the gold standard treatment to inhibit host proteases.^31^ According to Baca, et al.^32^ (2012), the effectiveness of CHX is related to concentration and frequency of application; the higher the concentration, the better the effect. For this reason, we sought a balance between concentration and frequency in this study to avoid side effects such as tooth staining or taste impairment.^33^ It has been shown that generalized tooth staining can occur if CHX is used in high concentrations and for a prolonged period.^34^ Several theories have been put forward to explain the cause of this characteristic staining, but most of the evidence indicates that staining is a result of the precipitation of anionic dietary chromogens (e.g., from tea, coffee, or wine tannins) onto adsorbed chlorhexidine cations. According to the literature, 0.12% chlorhexidine applications at time intervals between one and two minutes are unable to cause tooth staining,^35^ justifying the choice of this study.

The last treatment used in this study was the Nd:YAG laser, due to its ability to fuse and resolidify the substrate, acting on its organic and inorganic portions.^14 , 15 , 17^ In this treatment, hydroxyapatite is transformed into tricalcium B-phosphate, increasing the acid resistance of the tissue. This fact is relevant because, as reported in the literature, exposed dentine is more susceptible to cavities than enamel, and laser can increase the relative mineral content of the dentin.^25^ Thus, to obtain the desired effects via laser, the correct selection of parameters is fundamental.^36^ Energy density, power, frequency, and contact or not of the optical fiber with the substrate are certainly responsible for the great variability of results found in the literature. Therefore, the parameters selected for this study were 60 mJ of energy, 48 J/cm^2*^ of energy density, and 10 Hz of frequency, 100 μs in non-contact mode due to thermal melting and resolidification processes shown by laser irradiation in dentin.^37^ Nd:YAG wavelengths show reduced absorption by dental hard tissues and promote local, controlled temperature rises (less than 5.5ºC when 60 mJ is applied for 1 min in non-contact mode), leading to morphological and compositional changes in this substrate.^15 , 20^

Specimen storage in a collagenase solution provided collagenolytic activity after demineralization of the root dentin, also previously applied by Kato, et al.^7^ (2012) and Islam, et al.^6^ (2016). This methodology simulates the degradation of the carious process, in which, after demineralization in acid pH, the organic matrix is exposed to hydrolysis, accelerated by the enzymatic activity of host MMPs, cysteines, and cathepsins.^5^ Collagenase can promote “ *in vitro* ” degradation, releasing essential amino acids, which constitute collagen, in the storage solution. This release can be detected by colorimetric methods. Among these amino acids is hydroxyproline, which was analyzed in this study according to the literature.^6 , 7^

It is known that 90% of the dry mass of the DOM consists of type I collagen, which contains around 10% of HYP in its mass, whereas other proteins contain little or none of this amino acid.^38^ In our results, the assessed treatments were unable to reduce collagen degradation since they failed to decrease the amount of HYP released from the dentin samples, as Figure 2 shows. Thus, we accepted the 1^st^ null hypothesis since our treatments failed to reduce DOM degradation, as measured by the HYP assay.

Reddy and Enwemeka^39^ (1996) used the HYP assay and obtained positive results in their evaluation of degraded collagen in several biological tissues, proving, with their results, that the method is effective in tissues of different origins. Partially in agreement with Boteon, et al.^40^ (2017), our results were unable to differentiate the treatments, probably because we used the same clinical protocols for fluoride and CHX (i.e., 1-min applications). However, our results differed from previous studies^7^ that applied similar collagenase solutions. The authors induced a previous demineralization using another type of acid to simulate erosion (0.87 M citric acid, pH 2.3, for 36 h). Presumably, the amount of exposed DOM was higher in the case of erosion than those induced by cariogenic challenges, such as in this study, which may justify the differences. Based on these observations, we suggest that future studies should verify the influence of the demineralization degree and the thickness of the remaining DOM on the results of HYP release.

Our results are in agreement with Walter, et al.^41^ (2008), who also used the HYP method to evaluate collagen degradation in root dentin after treatments to promote the stability of the collagen matrix and prevent root cavities. Thus, it is important to stress that other forms of evaluation should be used to complement the analysis of how caries lesion progresses in dentin.

To calculate mineral loss and lesion progression between both demineralization challenges, we applied MRT since it is considered the gold-standard method to quantify the degree of demineralization and show the profile of the lesion.^25^ Even though we found no differences among the treatments, the groups that received the one-minute topical application of CHX (0.12%) or F (9000 ppm) showed a lower demineralization progress. Furthermore, CHX also reduced lesion depth progression after two additional days of demineralization.

Therefore, we reject our 2^nd^ null hypothesis. Based on our results, CHX was unable to decrease protease activity by HYP assay, as shown in previous works.^5^ However, it was able to reduce demineralization progression, which may be due to its precipitation on the surface, as shown previously.^12^ As it occurs for F (by CaF_2_ precipitates), CHX might also have some physical effect by occluding tubules of the dentin surface and protecting the tissue against further demineralization.

Even though this study was unable to detect low HYP release by chlorhexidine, the inhibitory effect of chlorhexidine on MMPs is attributed to a chelating mechanism since the inhibition of MMP-2 and MMP-9 could be prevented by the addition of calcium chloride binding chlorhexidine. It was also discussed how chlorhexidine might affect essential sulfhydryl groups and/or cysteine present in the active site of MMPs. At salivary concentrations above 0.2%, the inhibitory action of chlorhexidine might also relate to protein denaturation.^25^

Fluoride, on the other hand, proved to be effective in reducing mineral loss, but failed to affect lesion depth ( Tables 1 and 2 ). These results may relate to the fact that fluoride has a higher reaction on lesion surfaces, improving mineral content, even though it failed to penetrate sufficiently deep to impair or reduce acid penetration and lesion progression at its deeper portions.^29^

We observed that, regardless of the treatment, DOM removal failed to increase demineralization since the proposed protocol can remove unprotected collagen, but not its mineral content.^25^ However, when we compared results within the same group, we found no significant progression in lesion depth, except for the negative control without DOM compared to the control with DOM, showing that the presence of the DOM may decrease the depth of the acid penetration. On the other hand, CHX was the only treatment able to decrease lesion depth progression, which may be due to surface precipitation, as discussed above.

Despite the benefits of applying laser to promote morphological and/or chemical changes to the dentin surface,^14^ the induction of cracks and macroscopic voids may impair its action against demineralization, which justifies its lack of a protective effect in this study. Laser parameters are the key to obtaining good results with this technique. The literature shows huge differences between the protocols of laser application, making direct comparisons impossible.^14 , 20^ Thereby, studies should evaluate different parameters and/or the association of laser and fluoride or a photoabsorber to better understand the real contribution of this technology to the field. Future research should also conduct SEM images and chemical analyses to provide more information about the process.

Therefore, the field needs further studies to find the most adequate treatment, concentrations, treatment associations, and application times under models which simulate caries development under biofilm growth. Research should also explore the decreased lesion depth progression we observed in the CHX group and confirm our results under models closer to *in vivo* conditions.

## Conclusions

No treatment was able to reduce DOM degradation by HYP release. However, F and CHX reduced mineral loss progression, which is very promising when considering their clinical indication.
